# Case of Bronchocele

**Published:** 1830-06

**Authors:** Henry J. Raines

**Affiliations:** Member of the Royal College of Surgeons.


### BRONCHOCELE.

*Case of Bronchocele.* By HENRY J. RAINES, Esq. Member of the Royal College of Surgeons.

THE pathology of bronchocele is so little understood, and the physiological functions of the thyroid gland are so obscure, that I am induced to transmit the following case for insertion in your Journal; trusting that, ere long, some of my professional brethren, who have ampler opportunities than myself of investigating this disease, will be enabled to throw some light on these important and interesting points.

December 10th, 1829, Mrs. D., æt. thirty-seven, the mother of five children, applied to me on account of an enormous swelling, occupying the whole of the anterior and lateral parts of the neck; the superior portion ascending as high as, and nearly on a level with, the chin, and the inferior resting on the upper part of the sternum.

The patient states that she has been subject to glandular enlargements in the early part of her life, and particularly mentions one under the right knee, which having resisted various local and other remedies, a seton was passed through it by an active and intelligent surgeon, who then attended her; soon after which it entirely disappeared, and it was about this time she first observed the swelling in her neck.

It is now nearly ten years since this occurred, and the swelling has gradually and steadily increased in size, until it has attained its present great magnitude. The respiration is considerably affected, and at times impeded, and is accompanied by a peculiar wheezing; she talks very hoarsely, and is subject to flushing and tingling in the face. I cannot learn from her that the size of the tumor has varied at all during the periods of gestation, labour, suckling, or menstruation; or that it has been affected by the seasons, state of the bowels, &c. Pulse seventy; appetite good.

I prescribed for her ten drops of Tinct. Iodine, three times daily, a calomel purge twice a week, and an ointment containing one drachm of the Hydriodate of Potash, with an ounce of Axunge, to stimulate the surface of the tumor.

At the expiration of a fortnight, I found no perceptible alteration externally; the breathing, however, was much relieved, which led me to conclude that some pressure was taken off the trachea. The ointment could not be applied as I wished, as it was productive of pain and inflammation; I therefore substituted the Iodine ointment, and directed friction to be freely used with the hand. The dose of the Iodine was increased to fifteen, and subsequently to twenty drops three times a day, without the least interruption to or disorder of the bodily functions; and in the course of six weeks I had the satisfaction to see the tumor rapidly disappearing, and when, after having continued this treatment for three months, the patient left my neighbourhood, it was nearly entirely absorbed.

The length of time the tumor had existed, and the age of the patient, render this case worthy of notice. I do not remember to have met with an instance, although I have consulted the most eminent authorities, in which a cure was effected where the patient was above twenty-five years of age.

*Aldbrough, near Hull; April* 20, 1830.

